# War damages compensation: a case study on Ukraine

**DOI:** 10.12688/f1000research.136162.1

**Published:** 2023-09-29

**Authors:** Iryna Izarova, Yuliia Hartman, Silviu Nate

**Affiliations:** 1 Lucian Blaga University of Sibiu, Sibiu, Romania; 2 Taras Shevcheko National University of Kyiv, Kyiv, Ukraine; 3 Ivan Franko National University of Lviv, Lviv, Ukraine; 4 Global Studies Center, Sibiu, Romania

**Keywords:** reimbursement for damage; war damages compensation; war in Ukraine; settlement of disputes; transitional justice

## Abstract

Russia’s illegal, brazen and cynical full-scale invasion of Ukraine began on February 24th, 2022, and is still ongoing at the time of this research (July 2023). The damages incurred by Ukraine and its citizens during the years of occupation of the territories and the war are calculated in millions, although it is difficult to definitively determine both the methodology and specific numbers. To restore justice, it seems much more important to define a fair, transparent, and understandable procedure for compensating the losses suffered by citizens and businesses as a result of these events. This is especially important in the context of the need to implement the goals of sustainable development, in particular, ensuring equal access to justice for all. The article is devoted to these and related issues. To determine the procedure for compensating losses and damages caused by the war, we first determined what exactly can be compensated and who can apply for compensation. These and other factors determine the peculiarities of the procedure for the restoration of rights and compensation for damage caused by the war in Ukraine. In searching for an answer to the researched question, we analyzed the current legislation of Ukraine and draft laws proposed to regulate relations related to compensation for damages. We also conducted a comprehensive analysis of concepts such as losses, damages, compensation, reparations, and reimbursement as defined in national legislation and international treaties. The generalization of the case law of national courts (more than 200 analyzed decisions of the courts of the first and appeal and cassation instances for the period from February 20, 2014 to March 1, 2023, examples of which are presented in the study) indicates the presence of various approaches of compensation for damage, in understanding how to restore the violated rights of citizens.

## Introduction

«Difficult questions include who is included among the victims to be compensated, how much compensation is to be rewarded, what kinds of harm are to be covered, how harm is to be quantified, how different kinds of harm are to be compared and compensated and how compensation is to be distributed». ^1^

The tragedies of the war in Ukraine did not pass by anyone, at the same time, everyone’s situation is unique: different rights were violated, therefore, the damages that were incurred should be differentiated according to certain criteria.

Ukraine is obliged to provide victims with effective remedies (provide effective remedies to victims), namely to provide victims with equal access to effective judicial protection, as well as to other legal remedies, including access to administrative bodies and other bodies, as well as mechanisms, conditions and procedures in accordance with national legislation, which was defined by the UN in Basic Principles and Guidelines on the Right to a Remedy and Reparation for Victims of Gross Violations of International Human Rights Law and Serious Violations of International Humanitarian Law 2005. ^2^

Resolution of the Council of Europe ^3^ is foreseen (18) to create an international framework for compensating for any harm, loss, or injury caused. To realize this task, it was proposed the creation of an international register of damage, in cooperation with Ukraine and the EU member states aimed to keep records pertaining to evidence and claims regarding damage, loss, or injury suffered by individuals and entities, both natural and legal, in Ukraine (19.2). An international compensation commission was chosen as an option to examine and make decisions on the claims that have been submitted and documented through the registry. The second goal is to keep an indemnification fund that would disburse compensation awards to claimants who have successfully proven their claims (19.3). The main and the most interesting issue is how to incorporate the proposed ideas within the existing mechanisms of damage compensation according to the national laws of Ukraine.

This is especially important in the context of the need to implement the goals of sustainable development, in particular, ensuring equal access to justice for all. Goal 16 For Sustainable Development ^4^ emphasize the importance of robust and peaceful institutions that operate to guarantee equal access to justice for all individuals within inclusive and harmonious societies. Accomplishing this task amidst formidable obstacles such as the pandemic and the ongoing war in Ukraine was exceptionally challenging. Though Ukraine made some necessary steps forward ^5^ and considering mechanisms of war damage compensation should continue to keep introducing it. ^6^

In general, the principles underlying schemes for compensating victims of war are enshrined in international humanitarian law and international human rights law. These principles concern the protection and assistance of victims of armed conflict and the recognition of their right to compensation for damages incurred.

The compensation for damages can be realized in different ways and in different areas. *Financial compensation for material damages*: affected Ukrainian citizens could claim financial compensation for their homes, properties, and property destroyed during the conflict. Affected Ukrainian citizens who have lost their jobs or suffered economic losses due to the conflict could apply for emergency financial aid, economic reconstruction and revitalization programmes and other forms of support to restore their sources of income. *Medical and psychological assistance*: persons who suffered from violent crimes during hostilities could seek medical and psychological assistance to recover their mental and physical health. *Social aid and protection of children’s rights*: orphaned children and other vulnerable people could apply for social aid and protection of children’s rights to rebuild their lives and ensure they are not at risk of exploitation or abuse. *Humanitarian assistance and protection of refugees:* Ukrainian citizens who have had to take refuge in other areas of Ukraine or Europe could apply for humanitarian assistance and protection of refugees to provide shelter, food, water and different basic needs.

Having drawn attention to the above, in this article, we focus on the issues of compensation for the damage caused by Russia’s invasion of Ukraine, taking into account the specifics of other types of assistance to victims (such as medical, psychological, etc.), which are the subject of research in other fields of science.

The draft law proposed in March 2022 defined compensation for damaged and destroyed real estate during the war [ 1]. This does not mean that other types of losses caused by the war will not be compensated, but the purpose of this draft law was to define a simplified procedure for the restoration of rights, but at least other options for the restoration of violated rights will not be in the same conditions - simply and quickly.

In today’s difficult conditions of war and mass destruction, it is extremely important to ensure the achievement of the appropriate level of satisfaction of public expectations, when citizens suffering from war will receive fair compensation for their losses. Transitional justice is a complex phenomenon that has already existed on the territory of Ukraine over time since the temporary occupation of the territory of the East and Crimea, ^7^^–^^11^ which allows the introduction of flexible tools for restoring justice in post-war conditions.

As rightly emphasized in the Secretary General’s of United Nations report, published in August 2004, on the rule of law and transitional justice, in the conditions of massive violations of human rights, states are obliged to act not only against criminals, but also on behalf of victims - including by providing compensation, appropriate programs to compensate victims for the damage caused can to be an effective and operational complement by offering tangible remedies, fostering reconciliation, and reinstating victims’ confidence in the state, thereby acknowledging the valuable role of tribunals and truth commissions. ^12^ As stated in the last report, “designing and implementing inclusive, context-specific and victim-centered transitional justice processes” ^13^ is exactly the result we should strive for.

The experience of other countries clearly demonstrates the importance and urgency of providing a comprehensive approach to the settlement of the issue of compensation for the losses of the civilian population in war conditions, not limited to the introduction of separate procedures for the compensation of costs for the losses of this or that property.

In particular, the Main Legal Department of the Verkhovna Rada of Ukraine drew attention to the unreasonableness of limiting only compensation for damaged and destroyed immovable property, the correct remark about the selectivity of the mechanism cannot also be applied to all objects of civil rights provided for by the current legislation (Chapter 3 of the Civil Code). ^14^

We firmly believe, and our research will substantiate this standpoint, that the diverse array of losses endured by Ukrainian citizens and legal entities necessitates the pursuit of an effective and comprehensive mechanism for compensating damages. This mechanism should serve as an umbrella procedure encompassing the restoration of both property and non-property rights that were violated during the war and occupation.

We have to mention that in Ukraine, the institute of reimbursement of damages exists (‘vidshkoduvannia’), provided by Chapter 82 of the Civil Code of Ukraine, Articles 280, 515, 602, 1072, etc. In the meantime, the draft law on compensation consists of the other term, used in Ukrainian legislation in other ways. ^15^

The ways of protecting civil rights and interests include compensation for damages and other methods of reimbursement (‘vidshkoduvannia’) for property damage prescribed by Article 16 of the Civil Code of Ukraine. According to Article 22 of the Civil Code of Ukraine, a person who has suffered damages as a result of a violation of their civil right has the right to compensation; damages are: 1) losses experienced by an individual due to the destruction or harm to an object, along with the expenses incurred or to be incurred in order to rectify the infringement of their rights (real damages); 2) the potential earnings that an individual would have received under normal circumstances had their rights not been violated (forgotten benefit).

However, the concept of ‘compensation’ regarding compensation for property damage does not contain its definition or features. This definition is contained in the Law of Ukraine “On Copyright and Related Rights”, ^16^ in which it uses as well as definition “reimbursement”, but has some peculiarities.

The term “compensation” is also used in the Commercial Code of Ukraine, ^17^ the context of reimbursement of expenses for the purchase of machinery and equipment (Article 16, paragraph 2. The state may make compensations or additional payments to agricultural producers for agricultural products sold by them to the state).

At the same time, Article 152 contains provisions regarding the right of a business entity that carries out economic activity to demand compensation for damage caused to its natural resources by other entities. Part 1 of Article 225 of the Commercial Code contains provisions on possible material compensation for moral damage in cases provided for by law, which are part of the damages subject to compensation by a person who committed an economic offense. In a similar context, the concept is also used to determine the rights to compensation and reimbursement for losses to foreign investors (Article 397 of the Commercial Code of Ukraine).

Probably, the question of the correct application of the institutions of civil law and commercial law also lies in the realm of defining these two branches of law and the debates that take place in academic circles, ^18^^–^^21^ and may also be related to real problems of practical application since a separate branch of commercial courts operates in Ukraine. ^22^

Paradoxically, in Latin, the term “reimbursement” (‘vidshkoduvannia’) sounds like *compensatio.* It is also worth noting that the UN documents refer primarily to reparations. ^1^

As stated by the UN in the document Basic Principles and Guidelines on the Right to a Remedy and Reparation for Victims of Gross Violations of International Human Rights Law and Serious Violations of International Humanitarian Law 2005, in accordance with national legislation and international law, victims must be provided with full and effective compensation, which includes the following forms: restitution, compensation, rehabilitation, satisfaction and guarantees of non-repetition, ^2^ namely:

‘Restitution should, whenever possible, restore the victim to the original situation before the gross violations of international human rights law or serious violations of international humanitarian law occurred. Restitution includes, as appropriate: restoration of liberty, enjoyment of human rights, identity, family life and citizenship, return to one’s place of residence, *restoration of employment and return of property’ (authors’ italics).*

Among the specified forms is compensation, ^2^ which in content corresponds to reimbursement for damage under the legislation of Ukraine - this is compensation for any economically assessed damage, depending on the severity of the infringement and specific circumstances of each situation, that arise as a consequence of severe transgressions of international law, human rights, and significant breaches of international humanitarian law, such as: (a) Physical or psychological harm; (b) Foregone prospects, including employment, education, and social advantages; (c) Material losses and diminished income, including loss of future earning capacity; (d) Non-monetary harm; (e) Costs necessary for legal or expert aid, medications, medical services, as well as psychological and social support. Rehabilitation should encompass medical and psychological treatment, as well as legal and social assistance.

At the same time, science expresses different views on the possibilities of applying these institutions: as noted by Larry May, restitution is the return to the rightful owner of what was lost or taken, and reparation is the restoration in proper condition of what was damaged. Restitution and reparation have the same root, restoration, which itself is a type of correction or compensation (rectification or compensation). But each term emphasizes different aspects of the idea of recovery. May argues that when transitional justice is achieved, as opposed to ordinary compensatory justice, sometimes the person who caused harm is not the only one who has a duty to restore. ^23^

The question of the content of the concept is of great importance, since the appeal to the court with a demand for the protection of the right and the chosen method of protection of this right must comply with the current legislation, in particular, the main codified act regulating property and non-property relations - the Civil Code of Ukraine.

Questions regarding specific objects, losses, which are compensated under the specified Law, are dictated primarily by the desire to restore the rights of citizens who were left without a home, without a roof over their heads [ 2]. At the same time, any expenses that may have been caused by it should be reimbursed, otherwise, society’s expectations will not be fully met.

How to explain why the damaged house and not the car or other things are subject to compensation? Which are of interest to their owner. A house and an apartment are objects of property rights, like everything else; at the same time, it is precisely the aspect of their importance for the owner that should be decisive for assessing the value of compensation for expenses (photos, children’s personal clothes, etc.).

At the same time, in the conditions of war and transitional justice, it is not the fact of monetary compensation for losses that is important, but sometimes the very fact of recognizing these losses. Reparations may encompass non-financial aspects, as highlighted in the UN report, ^1^ such as reinstating victims’ legal rights, implementing victim rehabilitation programs, and adopting symbolic actions like official apologies, monuments, and commemorative ceremonies.

The absence of effective loss compensation mechanisms can lead to unexpected consequences, the experience of other countries of the world ^24^^–^^26^ confirms the need to introduce transparent and clear procedures, a defined procedure for compensation and a list of types of damage that are subject to them.

The looting of art objects by the Soviet Union and attempts to justify it are known to the whole world, ^24^ which should attract the special attention of scientists, primarily because one of the declared goals of the war is the destruction of the Ukrainian people and their culture, so in this case it is not about precious art objects as such, but about, in particular, objects of Ukrainian culture and art, decisive for the history and identity of the Ukrainian people [ 3].

It is worth paying special attention to the procedure for compensation for damage caused to the health and life of the Ukrainian and civilian population in general during the war. In particular, as emphasized ^25^ in a study of the Iraq and Afghanistan wars, the military paid out benefits to civilians for “accidental” injury, death, and/or property damage in order to “win” the hearts and minds of the population. The author reasonably concludes that while cash payments may alleviate short-term economic needs, the lack of legal accountability is problematic because it may contribute to increased impunity for combatant soldiers. ^26^ Therefore, not in every case of damage, compensation can be determined precisely in monetary terms.

As the UN report rightly points out, restoration of property rights, or simply compensation when this cannot be done, is a common problem, especially when done through massive government programs. Challenging inquiries arise regarding who is among the victims who should receive compensation, how much compensation should be paid, what types of damage are covered, how to quantify damage, how different types of damage are compared and compensated, and how compensation is to be distributed. ^1^

Simultaneously, one year has elapsed since the commencement of Russia’s active hostilities, yet no mechanisms have been developed to compensate losses or indemnify those who have suffered due to the war. In Ukraine, only the matter of compensating costs for the temporary accommodation (stay) of internally displaced persons remains regulated. ^27^ Persons who offered free accommodation to internally displaced individuals in Ukraine are eligible for this compensation, alongside employers who hired such individuals during the period of martial law in the country, and are entitled to reimbursement for their payment costs. ^28^

The examples of other countries make it necessary to carry out a more careful analysis of the possibilities of pre-trial or other alternative settlement of disputes related to the compensation of damage caused to citizens en masse [ 4]. Since this article does not analyze these issues in detail in view of the scope and goals of the research, it is important to emphasize the perspective of this direction for further research.

In particular, it is worth recalling that, according to the report, states should, among other things, make efforts to develop procedures that would allow groups of victims to submit claims for reparations and receive reparations, if appropriate. ^2^

Separate discussions are taking place in scientific circles regarding the compensation of ecological losses in Ukraine. ^29^ The experience of settling disputes on violation of the environmental rights of citizens and compensation for losses is quite demonstrative. In this regard, the UN Compensation Commission is a unique model of responsibility and compensation for environmental damage, which provided a legal process that cataloged, assessed and awarded funds to pay for the clean-up and restoration of damaged soil, water, coastal ecosystems and other damage caused by the war in the Persian Gulf 1990-1991 years. ^30^ The UNCC Environmental Program emphasizes the importance of an innovative approach to post-war justice. ^31^ The Commission adapted the traditional bilateral compensation commission model, which highlights the accountability of states for environmental harm during armed conflicts and underscores the advantages that can be derived from engaging in multilateral efforts and sustaining long-term commitments towards environmental restoration. ^32^

Among the issues that should be included in the circle of objects of compensation for damage caused by the war, the issue of relocation and return to the place of residence. As quite rightly noted in the literature, the issue of displacement during war is important, as we can see from the analysis of Ukrainian courts decisions, the results of which we present below, many displaced persons are in a legal limbo, without recognition and compensation. We also endorse the entitlement to return as recognized by customary international law, which obligates states to facilitate voluntary repatriation and provide restitution. ^2^ Facilitation entails refraining from obstructing or impeding the return process, preventing acts of retaliation or discrimination, and addressing the underlying causes of displacement. Restitution encompasses the restoration of property, compensation for both material and non-monetary harm, as well as measures promoting reintegration, reconciliation, and the implementation of effective return models. The study appropriately underscores the importance of determining the temporal aspect, the conditions for return, and addressing ongoing crises within the legal framework governing forced displacement. ^33^

In our opinion, it is not necessary to generalize the approaches to the definition of compensation as compensation for the costs of accommodation of internally displaced persons or for payment of employment, which are carried out by citizens voluntarily and on their own conviction, and compensation for damage caused by war, from which citizens suffer regardless of their will.

It is worth quoting here from the report that “no form of compensation is likely to satisfy the victims. Instead, as a complement to the proceedings of criminal tribunals and truth commissions, well-designed combinations of reparations measures are usually required.” As practice shows, the judges of the Yugoslavian and Rwandan tribunals recognized and suggested that the United Nations consider the possibility of creating a special reparations mechanism that would function alongside the tribunals. ^1^

Accordingly, the variety of losses suffered by citizens of Ukraine and legal entities prompts the search for an effective and multifaceted mechanism of reimbursement for damage, in the order of which various methods of legal protection can be applied, not only those provided for by the Civil Code of Ukraine, but also those referred to in UN reports - restoration of victims’ legal rights, victim rehabilitation programs and symbolic measures such as official apologies, monuments and commemorative ceremonies. One cannot and should not underestimate the attention and respect for the memory of the victims and losses suffered by the people of Ukraine and any other people from the burdens of the war, which cannot be compensated with money alone. At the same time, complex measures of recognition will perform another important function, a preventive one, with the aim of preventing possible similar conflicts in the future.

The main compensation procedure can be an umbrella procedure, within which the restoration of both property and non-property rights that were violated during the war and occupation will be carried out. At the same time, the application for compensation must be guaranteed for the loss of any objects of civil rights provided for by the current legislation, and not a list defined by law or in another order.

This study argues that the limitation or selective approach to determining the objects of compensation or expenses that are subject to reimbursement is incorrect and also violates basic human rights. The compensation mechanism should provide for a simplified and transparent version of the procedure for restoring the rights to housing, for resuming work, everything that ensures the possibility of a person’s normal life. At the same time, there should remain a real prospect of applying for the restoration of rights that have been violated in the person’s opinion, in particular, copyrights, or any others that cannot be classified as top priorities.

In this case, the actions that caused the violation of rights should be decisive - this is war and actions directly related to it (military actions, terrorist acts, sabotage caused by armed aggression). This will make it possible to clearly separate the actions and efforts aimed at restoring justice after the war, the expenditure of compensation funds.

What is the basis of the appeal and the reason for the violation of the right - war or occupation? Military actions or any actions during war? On the territory of all of Ukraine or on the territory of hostilities? The draft law on compensation of expenses states that it is about losses “as a result of hostilities, acts of terrorism, sabotage caused by the armed aggression of the Russian Federation against Ukraine”. ^34^ Analyzing court decisions reveals that the primary basis for compensation claims is Russia’s active armed aggression against Ukraine, which includes the occupation of certain Ukrainian territories, engagement in hostilities, acts of terrorism, and other related consequences that serve as valid grounds for such claims. On the other hand, the word “war” is not used either in judicial practice as a basis for going to court, or in national legal acts, that was discovered during the research of court decisions and national legislation, respectively.

As it was mentioned in Basic Principles and Guidelines on the Right to a Remedy and Reparation for Victims of Gross Violations of International Human Rights Law and Serious Violations of International Humanitarian Law 2005, ^2^ for compensation, the damage must be the result of gross violations of international law, human rights, and serious violations of international humanitarian law.

All these features make it necessary to return to the main question - which procedure should be used in case of incurring costs during wartime - reimbursement for damage or compensation? After all, in accordance with the Civil Code, reimbursement for damage is preceded by the determination of the composition of the civil offense, namely, who caused the damage, their illegal behaviour (action or inaction), the result and the cause-and-effect relationship between them. ^14^ At the same time, illegal behaviour is behaviour that does not meet the requirements of the law, entails a violation (reduction, limitation) of the property rights (goods) and legitimate interests of another person. In the conditions of war, it may not be so easy.

In order to prove the presence of damages, it is important to establish a causal relationship between the illegal behavior of the violator and the damages. The condition of responsibility for an offense is the fault of the person who caused the damage, that is, the guilty action is failure to perform, refusal to perform, or improper performance of obligations. The creditor, demanding reimbursement for damages, must prove the first three conditions of liability, in particular, the fact of the debtor’s illegal behavior, the amount of damages, and causation. The debtor’s guilt in the violation is presumed and cannot be proved by the creditor. Absence of fault is subject to proof by the person causing the damage.

At the same time, determining the procedure for reimbursement of expenses incurred in connection with the actions described in the law does not require compliance with such procedure and determination of the entire composition of the offense, since the law defines the conditions under which such reimbursement is paid. ^14^

The main method of protection of violated rights, provided for by the civil legislation of Ukraine, is reimbursement for the damage caused by the court procedure. In order to receive at least some satisfaction for the property and moral damage caused by the Russian Federation, which is certainly not able to fully end all the mental suffering of the persons who suffered from the act of armed aggression, the victims are applying to the court with claims for reimbursement for moral and property damage, caused by the armed aggression of the Russian Federation against Ukraine. ^14^

In connection with the ongoing full-scale invasion of the Russian Federation in Ukraine and realizing how much damage, both property and moral, has been caused during this time, ^35^ one can absolutely predict that the judicial system will experience a boom in claims for reimbursement.

This suggests that, in some cases, the compensation mechanism should be used to compensate war victims, so that in the process of restoring their rights, even greater trauma is not caused (for example, unjustified length of waiting for compensation, which is quite real in the case of judicial order of reimbursement).

The amount of compensation is also a question. According to the Basic Rules of the UN [ 5], ^2^ compensation must be provided for any economically evaluated harm that is suitable and commensurate with the gravity of the transgression and the specific circumstances of each individual case. This compensation should encompass lost prospects and the loss of potential earnings. States bear the responsibility to offer compensation to victims for acts or omissions attributable to the state itself, which amount to gross violations of international law, human rights, or severe breaches of international humanitarian law. However, there are known cases when national commissions significantly overestimated the amount of losses for real losses, and this led to a jump in economic growth. ^34^

Briefly summarizing, it is worth noting that efforts should be made by the war-affected state to implement a comprehensive compensation mechanism for reparations for victims of war, military operations, as well as including, but not limited to, ways of protecting rights provided for by national law, as well as special legislation on compensation for losses to war victims (non-property satisfaction measures for victims).

As regards the compensation plan for Ukraine, a coordinated strategy should be formulated to ensure that the Ukrainian citizens affected by the conflict with Russia receive the necessary help and assistance. This strategy should include the following:

‐ *Identification and registration of all persons affected by the conflict:* it is essential to identify and register all persons affected, including those who have fled or lost their jobs or property. Such data should be centralized in a register of victims of war to facilitate the provision of aid and assistance.
‐ *Assessment of the needs of the victims of war:* to be able to provide adequate help and assistance, it is crucial to assess the individual needs of the victims of war. This assessment should include medical, psychological, social and economic needs.
‐ *Providing appropriate assistance and help:* based on the assessment of the needs of the victims of war, proper assistance and aid should be provided. This could include financial support, training programmes, medical and psychological assistance, social protection and humanitarian aid.
‐ *Implementation of a system for compensating the victims of war:* The Government of Ukraine should implement a system of compensation for the victims of war to ensure that they receive adequate compensation for their material and economic losses.
‐ *Collaboration with international organizations:* Ukraine should work with international organizations such as the European Union, the United Nations and the Organization for Security and Cooperation in Europe to receive financial and technical support in providing aid and assistance to victims of war.
‐ *Promoting a culture of reconciliation:* To overcome the traumas of war and build a stronger and more united society, it is vital to encourage a culture of reconciliation and solidarity. This could include promoting intercommunity dialogue and cultural and educational exchanges between different ethnic groups in Ukraine.

It should be emphasized that the Ukrainian Government and the legislative power are actively working on the implementation of points of this generalized strategy. ^15^ Some of them (for example, implementation of a system for compensating the victims of war and collaboration with international organization in some aspects of compensation) are discussed in more detail in this article.

## Who can apply for compensation (reimbursement)?

«The purpose of compensation is to put the injured party in the same financial position they would have been in if the loss had not occurred. This compensation can include compensation for out-of-pocket expenses, lost income, pain and suffering, and other types of damages» [ 6]. ^2^

A fundamental question is who can apply for compensation if they believe their rights have been violated and costs can be reimbursed or compensated. An important issue is the protection of foreign investors, whose activities are crucial to the reconstruction of the state after the war. ^35^ From a practical perspective, the experience of Ukrainian legal entities and individual entrepreneurs in protecting rights violated during war can be helpful. However, the Ukrainian legal field does not yet have a compensation mechanism for damages caused by armed aggression to business entities. Therefore, the only way for these entities to obtain reimbursement is to go to court. ^17^ Since the beginning of the full-scale invasion, according to the data obtained on the web portal of the Unified State Register of Court Decisions, more than 100 business representatives (which, compared to the scale of destruction, is actually a small number) have applied to commercial courts with claims against the Russian Federation for damages caused to their property by enemy bombing, rocket and artillery fire. This has resulted in the destruction of both movable and immovable property and occupation, which has made it impossible to access the property and remove movable property while preserving its integrity. In addition to real losses, reimbursement is also available for lost profits due to the inability to carry out economic activities, especially due to occupation and destruction of property used for conducting such activities. Although, as a general rule, there is a presumption of the defendant’s fault in these cases, the plaintiffs (business entities) must substantiate their claims by indicating the circumstances under which their rights were violated and providing appropriate evidence to support the amount of damages to be recovered. This approach allows the business to receive the maximum possible amount of reimbursement proportional to the total amount of losses suffered (both real losses and lost profits) [ 7].

The conducted generalization shows that several categories can be distinguished among the natural persons who applied to the courts with claims for reimbursement for damages caused as a result of the armed aggression of the Russian Federation against Ukraine. The first category consists of *persons who have the status of a participant in hostilities and participated in the anti-terrorist operation (ATO) and/or the Joint Forces Operation (JFO).* Often, among such persons, those who were in captivity apply for reimbursement, which, of course, has a painful impact on the mental health of these defenders. We should not forget about physical injuries caused by combat wounds, which usually lead to a person receiving disability - such damage is also compensated by the courts at the expense of moral damages [ 8]. As judicial practice currently only knows cases of appeals for reimbursement by persons who participated in anti-terrorist operations, but there are still no examples of appeals with claims by persons who came to the defense of Ukraine at the beginning of a full-scale war (and an extremely large number of such appeals is expected). Then, in support of their demands, the participants of the ATO/JFO indicate, in addition to the circumstances of their captivity and the performance of combat missions, that after the beginning of the armed aggression of the Russian Federation against Ukraine, their general morale deteriorated, and their mental suffering intensified [ 9]. Similar examples of actions by participants in hostilities for reimbursement for moral damages can be seen in other court decisions (in 2022 [ 10] [ 11], and earlier, starting from 2016 [ 12]). It should be noted that judicial practice is moving towards the satisfaction of such claims.

The next category of plaintiffs that should be singled out are *family members of those killed as a result of the armed aggression of the Russian Federation.* A characteristic feature of this category of plaintiffs is that individuals seek reimbursement for moral damages related to the death of a family member, both on their own behalf and in the interests of minors, since in many cases, this deceased family member is the father of a minor child. In particular, the person applying to the court is the wife of the deceased, who acts on her own behalf and in the interests of her minor child and applies for reimbursement for moral damages from the Russian Federation. The basis for the lawsuits in this particular case is that the husband of the plaintiff and the father of her minor child, who was called up for military service as part of the Armed Forces of Ukraine, directly participated in the defense of the independence, sovereignty, and territorial integrity of Ukraine and died while performing a combat mission. The consequence of this and the damage caused is that due to the loss of her husband and the father of her child, the plaintiff experiences continuous, unrelenting mental pain and suffering, has lost peace of mind, constantly feels insecure and disappointed, and the child, left without a father, will never have the opportunity to feel the necessary parental love and attention for full development [ 13].

Adult children also file lawsuits for moral damages in connection with the death of one of their parents caused by armed aggression. Thus, in the mentioned cases, the plaintiffs, adult children, indicate that in connection with the death of one of their parents, they suffered moral damage, which consists of the fact that after learning about their parent’s death, they experienced strong emotional stress and a sense of mental pain from the awareness of the irreversibility of the loss of a loved one. Most of the plaintiffs were minors at the time of the death of one of their parents (mostly the father was a military serviceman), so they noted the loss of life orientations since, usually, the parental figure plays a significant role in the formation of a child’s personality and their upbringing. As a result of the loss, the minors had their usual way of life radically changed, and a depressed state was observed, which collectively caused them severe moral suffering. The courts satisfied such lawsuits, recognizing the Russian Federation as the cause and the sole culprit [ 14].

As an example of lawsuits regarding reimbursement for moral damage caused by the death of a family member, one can also cite the filing of such a lawsuit by one of the parents who lost a child - in the case cited as an example, the plaintiff filed a claim for reimbursement for moral damage caused in connection with the death of his son, who served in the Armed Forces of Ukraine and died while performing a combat mission. As a result of the death of his son, the plaintiff suffered moral damage, which consisted in the fact that he lost the meaning of life, suffered severe emotional stress, unrelenting mental pain and suffering, which will accompany him throughout his life [ 15].

Another category of plaintiffs in cases of reimbursement for moral and property damage are internally displaced persons who suffered damage to both property and moral well-being arising from the armed aggression carried out by the Russian Federation against Ukraine. The moral damage caused to internally displaced persons consists, for the most part, in the fact that, as a result of the occupation, these persons experience unbearable mental suffering due to the loss of housing, have lost the hope of returning home, a settled life, the opportunity to plan their lives, free communication with friends and relatives. In particular, due to the influence of Russian propaganda in the temporarily occupied territories, they are forced to deal with adaptation issues in a new place, which causes them severe stress and nervous breakdowns. They also suffer from a sense of longing for their native home, detachment from home, and the loss of the opportunity to pursue their previous activities and businesses [ 16]. In such cases, as experience shows, the court usually rules in favor of the injured person.

Among the cases on the reimbursement of moral damage, in which the plaintiffs are internally displaced persons, the cases based on the claims of persons who survived the occupation stand out especially in terms of their content. They impress with the detailing of the justification for the infliction of moral damage and the presentation of all the circumstances that the plaintiff had to experience during the occupation. The decision of the Bilotserkiv District Court of the Kyiv Region dated January 10, 2023 in case No. 357/9325/22 is telling, in which the court detailed all the circumstances of the case, which were referred to by the plaintiff, a resident of Mariupol, Donetsk Region. The plaintiff saw the beginning of a full-scale war in her native Mariupol and was under occupation for about a month. She described in detail the air raids and bombing of the city, the constant hiding in the basement of her own house, the difficulties of obtaining water and food, the lack of the possibility of performing basic hygiene procedures, being cut off from information, the destruction of the city, her way out of the occupied territory and the difficulty of passing the occupying checkpoints, and as well as her emotional experiences, fears and moral and physical suffering [ 17]. All these physical and moral sufferings were assessed by the plaintiff as moral damage caused to her by the Russian Federation and the court, agreeing with her arguments, satisfied the claims. In our opinion, such a clear statement of the circumstances of the case and the details provided by the plaintiffs are extremely important for making a decision on the satisfaction of the claims, especially when it comes to such a claim as reimbursement for moral damage, as shown by the analysis of judicial practice in this category of cases.

In this case, the applicant estimated the moral damage caused to her in the amount of UAH 1,000,000. While this assessment is not uncommon in judicial practice, it should be noted that this amount is unreasonable. In the majority of cases, plaintiffs determine the amount of moral damage in the hryvnia equivalent of 60,000 euros, which is determined according to the official exchange rate of the NBU as of the day of filing the claim (this amount, as of the time of the investigation, exceeds UAH 1,000,000). This technique is consistent with the practice of determining the amount of reimbursement for moral damage, which is reflected in the decisions of the ECHR [ 18]. Ukrainian courts consider these decisions as a source of law ^36^ and form their national law enforcement practice accordingly. If the monetary value of the moral damage exceeds the amount established by judicial practice, and there is no corresponding evidence to support such an amount, the court may partially satisfy such claims, to the extent that corresponds to the practice of the ECHR [ 19].

The category of internally displaced persons can be conditionally divided into *two subgroups - these are internally displaced persons who acquired this status before the start of a full-scale invasion on February 24, 2022, and, accordingly, after.* Therefore, court cases initiated by internally displaced persons can also be divided into two groups, which are characterized by different approaches to determining the amount of damage caused, as well as the origin of funds and property, at the expense of which reimbursement should be made.

In this regard, cases where plaintiffs also specified the origin of the property from which reimbursement should be made are interesting. Specifically, some plaintiffs requested reimbursement for damages caused by money obtained from forcibly seized property rights of the Russian Federation and its residents, or any other property belonging to the Russian Federation and located on the territory of Ukraine [ 20]. Regarding applications by internally displaced persons filed before February 24, 2022, a fairly straightforward formulation of the claim has been established, consisting of a specific amount of funds to be collected and the type of damage for which reimbursement is sought. However, the origin of the funds to be collected is not determined by the plaintiffs.

However, in such cases, the courts require sufficient evidence to confirm that the objects of property rights of the Russian Federation and its residents, or any other property of the Russian Federation, have been properly confiscated in Ukraine in accordance with the procedure established by law, and that the funds obtained from such confiscation can be used to compensate the moral damage caused to the plaintiffs. Unfortunately, such evidence is often not provided to the courts. The issue of forced alienation of objects of property rights of the Russian Federation and its residents on the territory of Ukraine or abroad is still subject to appropriate legal regulation and requires the adoption of appropriate decisions by state authorities [ 21]. Ukraine has confirmed this obligation in the resolution of the Cabinet of Ministers of Ukraine No. 326 dated March 20, 2022, “On approval of the Procedure for determining damage and losses caused to Ukraine as a result of the armed aggression of the Russian Federation”. ^37^ Therefore, based on the aforementioned considerations and legal groundlessness, the courts refuse to grant this part of the claim.

Regarding the property damage caused to internally displaced persons, in the analyzed case law, it is possible to distinguish between property damage caused by lost profits and real property damage caused by the loss of property (real damages). The lost profit can include the calculation of the cost of renting an apartment for the period during which the territory on which the property is located was under the occupation of the Russian Federation [ 22]. Additionally, plaintiffs include the deprivation of their right to use and dispose of the property, which is located in the temporarily occupied territory, belonging to them by the right of ownership, as a lost benefit in property damage [ 23]. The amount of property damage is measured using such indicators as costs associated with the forced rental of housing in the controlled territory of Ukraine, forced costs for housing maintenance (payment of communal services), etc. [ 24]. At the same time, the courts note that existing international and national normative legal acts do not contain provisions on the emergence of a temporarily displaced person’s right to reimbursement for the moral suffering suffered by them due to such displacement from the aggressor state [ 25]. We are of the opinion (and have already noted this in our research) that this approach of the courts is incorrect, and such damage should also be subject to reimbursement since the key factor in the displacement of a person is compulsion, which is connected with occupation, hostilities, terrorist acts, etc., that is, it is committed against the will of a person, which means that it causes them suffering.

Based on the analysis of judicial practice, there were cases where plaintiffs claimed compensation for property damage resulting from the armed aggression of the Russian Federation against Ukraine in the form of lost income due to forced displacement related to the occupation of part of Ukraine’s territory. The calculation of lost income due to forced displacement is determined from the moment of temporary occupation until the filing of the lawsuit by adding the annual average earnings of the specific position held by the internally displaced person before the forced displacement [ 26].

Regarding the real property damage, which is connected with the loss of property, internally displaced persons state in their lawsuits that their property was lost due to hostilities, terrorist acts, sabotage caused by the armed aggression of the Russian Federation, which violates the laws and customs of war. Accordingly, they ask the court to establish the legal fact of such destruction and oblige the Russian Federation to compensate for the damage caused. This should also include cases of non-fulfilment of contracts for the purchase and sale of property rights to immovable property located in the temporarily occupied territories of the Donetsk and Luhansk regions (TOT), which were not completed due to the armed aggression of the Russian Federation against Ukraine. According to such contracts, the seller-developer was obliged to complete and transfer ownership of the home to the buyer-plaintiff. Such situations occurred in 2014, when Russia began its aggression against Ukraine by occupying Crimea and the Donetsk and Luhansk regions. It is clear that at that time no property was built under such contracts and no one returned any money to the buyers, so the buyers demanded reimbursement for the damages caused by Russia in court [ 27].

We should also not forget about property damage in the form of the loss of movable property, which became especially relevant with the beginning of the full-scale invasion and mass robberies of Ukrainian homes by Russian servicemen. For example, in one case, the plaintiff stated that Russian servicemen robbed her apartment with particular disdain, cynicism, and cruelty. They destroyed all the furniture, appliances, and other things in the apartment and also stole her car. However, in many cases, as in this case, it is impossible to assess the value of all the stolen property due to the Russian occupation. Therefore, the plaintiff asked for reimbursement for the damage caused by the theft of only the car, assessing its value based on the publicly available information about the value of a car of the same model [ 28]. Among the movable property that was lost by internally displaced persons as a result of hostilities and occupation, personal belongings are also mentioned, which, although they do not have great property value, their sentimental value cannot be determined from the point of view of the significance of these things for such persons. These things carry memories and personal history. The practice is that the loss and/or destruction of things that are sacred to their owner is assessed by the plaintiffs themselves as moral damage caused to them, and the courts agree with this assessment.

In cases of reimbursement for moral damage caused as a result of Russia’s armed aggression against Ukraine, it is worth highlighting another category of plaintiffs such as ordinary citizens of Ukraine. These individuals justify their legal claim by stating that since the beginning of the armed aggression of the Russian Federation against Ukraine, they have suffered mental distress, their well-being has deteriorated, and their vitality has decreased. This is due to daily news about the death and injury of people, explosions and destruction of buildings, taking and torturing hostages in the war zone, witnessing funeral processions for the fallen defenders of Ukraine, and awareness of the scale of destruction of the economy and ecological disaster in the territories where the hostilities took place. Courts usually reject such claims due to the lack of evidence of actual moral damage, apart from the plaintiffs’ generalized statements [ 29]. However, in practice, there are sometimes cases where courts make a positive decision under such circumstances [ 30]. In such cases, it is important not only to establish the fact of armed aggression against Ukraine, which in itself causes mental suffering to every conscious citizen, but also to confirm such suffering and its specific manifestations in each individual situation.

## Who is responsible for compensation?

«16. States should endeavour to establish national programmes for reparation and other assistance to victims in the event that the parties liable for the harm suffered are unable or unwilling to meet their obligations». ^2^

Given that states bear the responsibility of safeguarding the rights of their citizens, encompassing the rights to life, physical well-being, and property, the allocation of accountability for inaction, and subsequently the state’s obligation to finance reparation initiatives, can be significantly burdensome. While states should ideally hold the individuals directly responsible for the wrongful harm liable for restitution, if this proves unfeasible or inadequate in achieving the goal of reparation, then the state itself assumes responsibility. This means that taxpayers, and perhaps their future generations, must bear the burden of recovery. ^38^

Russia is responsible for the damage caused during the war with Ukraine. Relevant international regulations that can be applied in this situation include the Hague Convention on the Laws and Customs of Land Warfare of 1907, ^39^ the Geneva Convention for the Treatment of Prisoners of War of 1949 ^40^ and Additional Protocol I to the Geneva Convention of 1977. ^41^ They set international standards on the treatment of civilians, prisoners of war and civil property during armed conflicts.

Ukraine could also invoke the provisions of the Convention on the Protection of Human Rights and Fundamental Freedoms, which provides for the right to life, the right to respect for private and family life and the property right. ^42^ These rights were violated during the war, and Russia is obliged to provide adequate compensation.

Based on the generalization of Ukrainian judicial practices, it can be observed that in the majority of cases, the defendant is the Russian Federation. The plaintiffs address their claims for reimbursement either to the Russian Federation, or to the Russian Federation in the person of the Embassy of the Russian Federation in Ukraine or the Ministry of Justice of the Russian Federation. The plaintiffs justify the Russian Federation’s defendant status by stating that it is responsible for violating the rights and freedoms of citizens of Ukraine, including the personal rights of the plaintiffs, as a result of its armed aggression and occupation of Ukrainian territory. Therefore, the Russian Federation is the entity charged with the obligation to reimburse for the damages caused by these actions.

However, from the perspective of international law, the question arises regarding the legality of prosecuting the Russian Federation as a foreign state. According to the general rule established by the Vienna Convention on Diplomatic Relations of April 18, 1961, ^43^ to which Ukraine is a party, foreign states are entitled to judicial immunity, which means that they cannot be subjected to the legal authority of the courts in a different state without their explicit or tacit consent. Thus, each sovereign state can only participate in a court case in another state with its consent expressed through authorized persons. This position was also stated in the decision of the Supreme Court dated September 1, 2021, in case No. 754/10080/19 [ 31]. Therefore, the usual practice of Ukrainian courts, in accordance with the Regulation on Diplomatic Missions and Consular Institutions of Foreign Countries in Ukraine, approved by the Decree of the President of Ukraine No. 198/93 of June 10, 1993, ^44^ is to send a letter through the Ministry of Foreign Affairs of Ukraine to the Embassy of the Russian Federation requesting consent to consider the case and granting the Russian Federation the procedural status of the defendant in the case. It should be noted that letters of this nature were typically sent several times as the Russian side did not reply due to its non-recognition of its acts of armed aggression, the illegal annexation of the Autonomous Republic of Crimea, and the occupation of the eastern regions of Ukraine. Such ignoring is regarded by the courts as giving the defendant tacit consent to participate in the case by its actions, as provided by the Law of Ukraine “On International Private Law”. ^45^

In addition, the courts take into account the violations of international law committed by the Russian Federation. The courts believe that the Russian Federation, by violating the UN Charter, ^46^ the Universal Declaration of Human Rights, ^47^ the Budapest Memorandum (paragraphs 1 and 2), ^48^ the Helsinki Final Act of the Conference on Security and Cooperation in Europe dated August 1, 1975, ^49^ as well as the agreements concluded between Ukraine and Russia, including those related to the Ukrainian-Russian state border, has exceeded its sovereign rights, which are guaranteed by Article 2 of the UN Charter. Therefore, the Russian Federation is considered an aggressor state according to the normative acts adopted by Ukraine, and is therefore not entitled to judicial immunity.

Jurisprudence regarding the absence of judicial immunity in the Russian Federation and the legality of its prosecution by Ukrainian courts had been developing in the direction described above until the Supreme Court formulated a new conclusion regarding the judicial immunity of the Russian Federation in the case of reimbursement for damage caused by the aggressor state in 2022 [ 32].

The Supreme Court referred to the European Convention on the Immunities of States, adopted by the Council of Europe on May 16, 1972, and the UN Convention on the Jurisdictional Immunities of States and their Property, adopted by resolution 59/38 of the General Assembly on December 2, 2004, which reflect the evolving trajectory of international law, acknowledging that foreign states have certain limitations on claiming immunity in civil proceedings. The court concluded that a state cannot invoke immunity in cases involving harm to health or life if such harm is wholly or partially caused within the territory of the court’s state and if the person responsible for the harm was present in the court’s state at that time. The Supreme Court further highlights the principle of “equality among equals” in terms of power and jurisdiction, which necessitates mutual recognition of sovereignty among nations. Since the Russian Federation denies the sovereignty of Ukraine and engages in aggressive warfare against it, there is no obligation to respect and uphold the sovereignty of Ukraine.

The court reached the determination that, given the continuous and extensive armed aggression perpetrated by the Russian Federation against Ukraine, which flagrantly infringes upon its sovereignty, it is presently unsuitable to seek the consent of the Russian Federation to participate as a defendant in such cases. Halting the proceedings to secure the Russian Federation’s consent would result in an unjustifiable delay in the case’s adjudication, thus failing to serve the best interests of the plaintiff in terms of safeguarding their rights effectively.

As a consequence of the unprovoked and extensive act of armed aggression by the Russian Federation against Ukraine, along with numerous acts of genocide perpetrated against the Ukrainian people, the Russian Federation will no longer be eligible to invoke judicial immunity. Consequently, Ukrainian courts are empowered to consider and resolve cases seeking reimbursement for damages arising from such acts of aggression. The Supreme Court asserts that the aggressor country, in question, did not operate within the boundaries of its sovereign right to self-defense but instead deliberately violated all sovereign rights of Ukraine while conducting its actions on Ukrainian territory. Hence, the Russian Federation unequivocally forfeits the privilege of utilizing judicial immunity in this specific category of cases [ 33].

The analyzed court decisions revealed cases where, prior to the formulation of the Supreme Court’s aforementioned positions [ 34] the courts denied claims for reimbursement for damages caused by the Russian Federation’s armed aggression against Ukraine. This was due to an incorrect interpretation of the judicial immunity of the Russian Federation, which was mistakenly considered absolute without taking into consideration the evolution of international law ^50^ and the specifics of cases related to reimbursement for damages. Such cases allow exceptions under which a foreign state can be prosecuted. The courts also misinterpreted the tacit consent of a foreign state to participate in the legal process. ^51^

In particular, some court decisions noted that “despite the temporary occupation of Ukrainian territories resulting from the armed aggression of the Russian Federation, it remains unchanged that the Russian Federation, as a sovereign state, cannot be a party to a case in a general jurisdiction court of Ukraine without its explicit or implied consent expressed through its authorized entities or officials.” The courts considered the potential ramifications, including the non-recognition and non-enforcement of Ukrainian national court decisions by the Russian Federation [ 35]. The evident outcome of such refusals was an appeal, and later a cassation appeal, of court decisions, resulting in the plaintiffs’ demands still being subject to satisfaction [ 36].

The Unified State Register of Court Decisions contains cases of reimbursement for damage caused by the armed aggression of the Russian Federation against Ukraine, in which the defendant is not only the Russian Federation as a foreign state, but also the Embassy of the Russian Federation in Ukraine, represented by the Ministry of Justice of the Russian Federation, and JSC “Sberbank”, which was later renamed to JSC “International Reserve Bank” and thus became jointly liable with the Russian Federation for reimbursement. However, there is a lack of consistency in judicial practice regarding whether JSC “International Reserve Bank” can be recognized as a proper defendant in these cases. In case No. 215/294/21, for example, the court ruled that there was no legal basis for collecting moral damages from the Bank as the plaintiff failed to provide evidence of which actions of the Bank caused damage and violated their rights. The court concluded that the entire burden of reimbursement rests solely on the Russian Federation, as the sole guilty party [ 37]. We agree with such conclusions as establishing guilt and a causal relationship between the subject’s actions and the damage is necessary to establish the subject’s obligation to compensate for the damage caused. Although some courts recognize JSC “International Reserve Bank” as a proper defendant [ 38], such conclusions are later found to be erroneous during the appeal process [ 39].

Ensuring the unity of judicial practice and observance of human rights is an important task. The generalization of the national judicial practice during the 9 years of the armed aggression of the Russian Federation against Ukraine - from 2014 to 2022 - indicates the presence of a basic practical framework, in particular, in the justification by the courts of the motives from which they proceed when making a decision. This observation concerns both courts of first instance and appeals courts, and, of course, the Supreme Court, which, in fact, forms the unity of judicial practice and the application of legal norms by courts.

In the motivational part of the court decision, the courts paid considerable attention to justifying the legality of the participation of the Russian Federation as a party in civil proceedings and its recognition as a proper defendant due to the presence of its judicial immunity. Although, after the Supreme Court formulated its legal position regarding the lack of judicial immunity of the Russian Federation [ 40] in the studied category of cases, this necessity disappeared. We have investigated in detail this issue and the motives from which the courts proceeded both in the period from February 20, 2014 to February 24, 2022, in the first two phases of the armed aggression of the Russian Federation against Ukraine, and after the full-scale invasion that began on February 24, 2022, and continues to this day. However, for research purposes, it is important to find out what other arguments the courts use to strengthen their position when satisfying or refusing to satisfy claims.

First of all, it is important to ascertain a cause-and-effect relationship between the actions of the defendant (in our case, the Russian Federation) and the damage that was caused, whether moral or property, for the success of the claim. Courts during the trial are obliged to fully, comprehensively and impartially investigate all available evidence in the case in order to establish this cause-and-effect relationship, and therefore the validity of the satisfaction of the claims. Therefore, before the courts when considering cases of reimbursement for damage caused as a result of the armed aggression of the Russian Federation against Ukraine, the primary task is to establish the relationship between the armed aggression of the Russian Federation and the damage that was caused.

When satisfying claims for reimbursement for moral and property damage caused as a result of the armed aggression of the Russian Federation against Ukraine, the courts proceed from this.

According to Part 4 of Article 2 of the Law of Ukraine “On the Legal Status of the Temporarily Occupied Territory of Ukraine,” the responsibility for any material or non-material harm inflicted upon Ukraine due to the armed aggression by the Russian Federation lies with the Russian Federation, in accordance with the principles and standards of international law [ 41]. ^52^ Part 6 of Article 5 of the same law corresponds to the above-mentioned norm, which is also referred to by the courts in their decisions. The statement asserts that the responsibility for compensating for both tangible and intangible harm incurred due to the temporary occupation falls entirely on the Russian Federation, acting as the occupying nation, in relation to the state of Ukraine, legal entities, public associations, Ukrainian citizens, foreigners, and stateless individuals. ^53^

The courts also refer to the Resolution of the Verkhovna Rada of Ukraine No. 145-VIII, dated February 4, 2015, “On Ukraine’s recognition of the jurisdiction of the International Criminal Court regarding the commission of crimes against humanity and war crimes by high-ranking officials of the Russian Federation and leaders of the terrorist organizations “DPR” and “LPR” which led to particularly grave consequences and the mass murder of Ukrainian citizens” when forming the motivational part of the decision. This resolution states that since February 20, 2014, the armed aggression of the Russian Federation against Ukraine has been ongoing, and the terrorist fighters supported by it have occupied part of the Donetsk and Luhansk regions of Ukraine and annexed the Autonomous Republic of Crimea and the city of Sevastopol, which are part of the territory of the independent and sovereign state of Ukraine. The resolution also highlights that numerous Ukrainian citizen, including children, have lost their lives, while thousands have sustained injuries. Moreover, the resolution emphasizes the extensive destruction of the entire region’s infrastructure, and the displacement of hundreds of thousands of citizens from their homes. The courts refer to this resolution to establish the background and context of the armed aggression and to emphasize the gravity of the consequences of the actions of the Russian Federation. ^54^

The courts have used normative legal acts to support the assertion that the Russian Federation has been committing armed aggression against Ukraine since 2014. This reasoning has been repeatedly upheld in court decisions that have awarded damages in cases related to full-scale invasion [ 42]. As a result, the courts now routinely refer to the Russian Federation’s aggression against Ukraine as a well-established and generally recognized fact. There is no longer a need for a detailed description of the legal acts that confirm this, as the international legal community and Ukrainian judicial practice have both recognized Russia’s aggression against Ukraine.

The motivational part of court decisions related to reimbursement for damage caused by the Russian Federation’s armed aggression against Ukraine has evolved with the onset of the war and the expansion of both national and international legal frameworks. Specifically, recent decisions have included references to a resolution adopted by the Verkhovna Rada of Ukraine on April 14, 2022, which recognizes the actions of the Russian Armed Forces and political and military leadership as genocide against the Ukrainian people. This resolution was passed in response to the ongoing armed aggression by Russia against Ukraine, which began on February 24, 2022. ^55^

Furthermore, courts have cited the resolution on the termination of the Russian Federation’s membership in the Council of Europe as evidence in their decisions [ 43]. This resolution affirms that the Russian Federation’s aggression against Ukraine is a grave violation of its obligations under Article 3 of the Charter of the Council of Europe.

When substantiating the size and necessity of reimbursement for moral damage caused, courts have cited the decision of the European Court of Human Rights in the case of “Luizidou v. Republic of Turkey” [ 44]. This case involved the illegal occupation of the northern part of Cyprus by the Turkish Armed Forces, where the applicant was born and lived. The Court ruled that the Republic of Turkey was obliged to pay compensation to the applicant, including for moral suffering resulting from the occupation. The Court found that the applicant had suffered psychological harm, including forced relocation from her home, an inability to live in the occupied territory, and a feeling of fear and helplessness. This decision has been used as a precedent for determining reimbursement for moral damage caused in cases related to the Russian Federation’s armed aggression against Ukraine.

When determining the amount of reimbursement for moral damage caused to the applicant, Ukrainian courts rely on the amount of claims that have been granted in similar disputes considered by the European Court of Human Rights. For instance, in the case of “Luizidou v. Republic of Turkey” [ 45], the European Court of Human Rights ruled that the Republic of Turkey had to pay the applicant compensation in the amount of 20,000 Cypriot pounds. At the time of the dispute in the European Court of Human Rights, which was prior to the introduction of the euro, the European Union used the exchange rate of 0.585274 Cypriot Pounds to 1 Euro [ 46].

In addition, when addressing this matter, courts take into consideration relevant rulings by the European Court of Human Rights in cases such as “Khachukayeva v. Russia (23/04/2009)” [ 47], “Sagayeva and Others v. Russia (08/12/2015)” [ 48], “Islamova v. Russia (30/04/2015)” [ 49], and “Sultygov and Others v. Russia (09/10/2014)” [ 50].

In addition, under international law, Ukraine can claim compensation for damage caused by Russia under the principle of international state responsibility. Such damage should include material damage, moral damage to the loss of human life, the victims’ physical and psychological suffering, and the destruction of civil property.

As of today, Ukraine is actively working on collecting the evidence base for future trials to hold Russia accountable for what it has done, including the recovery of compensation, as well as legal mechanisms for creating special courts and addressing already existing international courts. In today’s conditions, these issues are extremely important and promising for further research.

On the other hand, next to the question of determining the procedure for bringing the perpetrator to justice for the damage caused and the obligation to compensate for it, the question of exactly by what means this damage can be compensated arises. One of the options for sources of compensation, which is discussed in society, is “frozen” funds and other property of Russia and its citizens in Western countries. However, this issue also should to be studied additionally and has researching perspective for future works.

## Conclusion

The diversity of losses suffered by Ukrainian citizens and legal entities during the aggression of the Russian Federation since 2014 necessitates the search for an effective and multi-option mechanism for compensating damages. In our opinion, such a mechanism should be a comprehensive ‘umbrella’ procedure that provides opportunities for the swift and cost-effective consideration of claims for reimbursement for various types of damage and losses in different categories of cases. As the analysis of existing approaches and the generalization of judicial practice show, there are specific issues regarding reimbursement for material and moral damages, depending on the composition of the case.

The generalization of judicial practice in cases of reimbursement for damage caused as a result of Russia’s armed aggression against Ukraine has allowed the identification of categories of persons who have applied or are continuing to apply for the protection of their rights that were violated by the war, including family members of deceased persons (most often military personnel), participating military personnel in hostilities, and internally displaced persons. It has become evident that the plaintiffs, as well as the Ukrainian courts, maintain a united and firm position by naming the Russian federation as the defendant in this category of cases. The main demands that are unanimously put forward to the aggressor state are reimbursement for moral and material damages. However, despite the well-defined position of the overwhelming majority of courts, including the Supreme Court, there are cases where courts of appeal and cassation review decisions based on complaints from injured persons due to incorrect application of the law by courts of first instance.

The impact of the European Court of Human Rights’ practice on national judicial practice and the unity of approaches that have emerged since 2014 indicate the grounds for the introduction of simplified procedures for determining compensation and its recovery, particularly in cases of moral damage caused by armed aggression against Ukraine. More accessible and unified procedures will also aim to relieve claimants of exhausting court proceedings and additional stress. The fact of armed aggression by the Russian federation against Ukraine is rightfully considered universally recognized, meaning that it does not require further evidence, which creates additional opportunities for simplifying and improving the protection procedures of the affected parties.

The adopted resolution of the Council of Europe provides significant impetus for further searching and implementing mechanisms for compensation for the losses suffered, as well as for the establishment of a register of losses. The resolution establishes specific guidelines that must be considered when formulating and implementing procedures for compensating the damages resulting from military actions. However, at the same time, the issue of compensating for the damage caused by the occupation of Ukrainian territory since 2014 should not be overlooked, and it also needs to find a proper place in the mechanism that ensures equal access to justice for all who have suffered.

The fact that decisions have been made to recover funds from the Russian Federation government means that it will be necessary to search for property and means of recovery, which will be a challenging process. This may be the aim of further studies.

## Data Availability

All data underlying the results are available as part of the article and no additional source data are required.

## Footnotes

^1^
The bill on compensation of expenses was introduced in 2022 by the President of Ukraine. Available at: https://itd.rada.gov.ua/billInfo/Bills/Card/39283. It was adopted by the Verkhovna Rada of Ukraine on February 23, 2023. Available at: https://zakon.rada.gov.ua/laws/show/2923-20#Text. On March 17, 2023 the Law was signed by the President of Ukraine and is entering into force.

^2^
Even the loss of real estate should be assessed more comprehensively, taking into account the destruction of the surrounding infrastructure, see Al-Harithy H., *Lessons in Post-War Reconstruction: Case Studies from Lebanon in the Aftermath of the 2006 War*, *Lessons in Post-War Reconstr.: Case Stud. from Leban. in the Aftermath of the 2006 War*, Lessons in Post-War Reconstruction: Case Studies from Lebanon in the Aftermath of the 2006 War (Taylor and Francis, 2010), https://doi.org/10.4324/9780203851524.

^3^
Regarding the specifics of the known experience, cultural objects took a prominent place in reparations, which is now interesting to rethink through the prism of property rights. Between 1919 and 1945, there were two contrasting cases where the legal status of cultural objects changed through reparations mechanisms. See details Perrot X., ‘ *Cultural objects in the reparation for war damages, 1919 and 1945, restitutio in integrum (restoration to original condition), compensation and fungibility*’, Revue Historique de Droit Francais et Etranger 84, no. 1 (2006): 47–69.

^4^
It concerns the environmental rights of citizens, in particular see the case in more detail Jobin P., ‘ *The Economy of Compensation and Struggle for Reparation: The Case of Formosa Plastics in Taiwan*’, in Rethinking Post-Disaster Recovery: Socio-Anthropological Perspectives on Repair. Environments (Taylor and Francis, 2021), 25–48, https://doi.org/10.4324/9781003184782-3.

^5^
Compensation of damages refers to the monetary compensation awarded to a person or entity as a result of losses or harm they have suffered as a result of a breach of contract or tort (civil wrong).

^6^
Compensation of damages refers to the monetary compensation awarded to a person or entity as a result of losses or harm they have suffered as a result of a breach of contract or tort (civil wrong). The purpose of compensation is to put the injured party in the same financial position they would have been in if the loss had not occurred. This compensation can include compensation for out-of-pocket expenses, lost income, pain and suffering, and other types of damages.

^7^
See more details in the Decision of the Commercial Court of the Kyiv region dated January 30, 2023 in case No. 911/2065/22 (Available at: https://reyestr.court.gov.ua/Review/108985076), the Decision of the Commercial Court of the Kharkiv region dated October 18, 2022 in case No. 922/916/22 (Available at: https://reyestr.court.gov.ua/Review/106939659), the Decision of the Commercial Court of the Kyiv City dated December 6, 2022 in case No. 914/1552/22 (Available at: https://reyestr.court.gov.ua/Review/107702941).

^8^
The Decision of the Baryshiv District Court of the Kyiv Region dated November 11, 2019 in case No. 355/1030/19. Available at: https://reyestr.court.gov.ua/Review/85591782.

^9^
The Decision of the Dovhyntsiv District Court of the city of Kryvyi Rih, Dnipropetrovsk region in case No. 211/3600/20. Available at: https://reyestr.court.gov.ua/Review/103890683.

^10^
The Decision of the Khmelnytskyi City and District Court of the Khmelnytskyi Region dated September 12, 2022 in case No. 686/21759/21. Available at: https://reyestr.court.gov.ua/Review/107478121.

^11^
The Decision of the Central City District Court of Kryvyi Rih, Dnipropetrovsk Region dated September 22, 2022 in case No. 216/3586/21. Available at: https://reyestr.court.gov.ua/Review/106871522.

^12^
The Decision of the Yarmoline District Court of the Khmelnytskyi Region dated December 14, 2016 in case No. 689/1880/16-ц. Available at: https://reyestr.court.gov.ua/Review/63701174.

^13^
The Decision of the Leninsky District Court of Kirovohrad of December 4, 2018 in case No. 405/6702/18. Available at: https://reyestr.court.gov.ua/Review/78297243. Similar cases of filing claims for reimbursement for moral damages by plaintiffs who are family members of persons killed as a result of armed aggression against the Russian Federation can be traced in the vast majority of investigated cases to the Decision of the Uzhhorod City District Court of Zakarpattia Oblast dated February 2, 2021 in case No. 308/9708/19, the Decision of Zhovtnevy of the District Court of Kryvyi Rih from June 21, 2022 in case No. 212/4039/21 (Available at: https://reyestr.court.gov.ua/Review/104854655), the Decision of the Khmelnytskyi City and District Court of the Khmelnytskyi Region dated November 13, 2018 in case No. 686/21800/18 (Available at: https://reyestr.court.gov.ua/Review/77861694).

^14^
The Decision of the Terniv district court of the city of Kryvyi Rih, Dnipropetrovsk region, dated June 8, 2022, in case No. 215/294/21. Available at: https://reyestr.court.gov.ua/Review/104683338.

^15^
The Decision of the Saksagan District Court of Kryvyi Rih, Dnipropetrovsk Region dated May 18, 2022 in case No. 214/5601/21. Available at: https://reyestr.court.gov.ua/Review/104420460. Similar claims and circumstances of the case are contained in the Decision of the Brovary City and District Court of the Kyiv Region dated September 5, 2017 in case No. 361/2955/17 (Available at: https://reyestr.court.gov.ua/Review/69248932), the Decision of the Derazhnyan district court of the Khmelnytsky region dated December 21, 2017 in case No. 673/1550/17 (Available at: https://reyestr.court.gov.ua/Review/71328444), the Decision of the Rivne city court of the Rivne region dated November 30, 2018 in case No. 569/18381/18 (Available at: https://reyestr.court.gov.ua/Review/78434066). Courts usually decide such cases in favor of the plaintiffs.

^16^
The Decision of the Starobil district court of the Luhansk region dated December 27, 2018 in case No. 431/4115/18. Available at: https://reyestr.court.gov.ua/Review/78392525. Similar cases and justifications are given in other decisions of national courts, in particular, in the Decision of the Supreme Court as part of the panel of judges of the First Judicial Chamber of the Cassation Civil Court dated May 18, 2022 in case No. 428/11673/19, the Decision of the Central City District Court of of Kryvyi Rih, Dnipropetrovsk region from October 12, 2022 in case No. 216/1907/22 (Available at: https://reyestr.court.gov.ua/Review/106996083), the Decisions of the Khmelnytskyi City and District Court of July 25, 2022 in case No. 686/30265/21 (Available at: https://reyestr.court.gov.ua/Review/106416818).

^17^
The Decision of the European Court of Human Rights in the case of *Louisidou v. Republic of Turkey (28/07/1998).* Available at: https://hudoc.echr.coe.int/ukr#{“tabview”:[“document”],“itemid”:[“001-58201”]}.

^18^
The Decision of the Bilotserkiv District Court of the Kyiv Region dated January 10, 2023 in case No. 357/9325/22. Available at: https://reyestr.court.gov.ua/Review/108371522.

^19^
The Decision of the Central City District Court of Kryvyi Rih, Dnipropetrovsk Region dated April 9, 2021 in case No. 216/4601/20. Available at: https://reyestr.court.gov.ua/Review/96315393.

^20^
The Decision of the Ivano-Frankivsk City Court of the Ivano-Frankivsk Region dated July 26, 2022 in case No. 344/5555/22. Available at: https://reyestr.court.gov.ua/Review/105546152.

^21^
The Decision of the Ivano-Frankivsk City Court of the Ivano-Frankivsk Region dated July 26, 2022 in case No. 344/5555/22. Available at: https://reyestr.court.gov.ua/Review/105546152. The same conclusions are contained in the Resolution of the Grand Chamber of the Supreme Court dated May 12, 2022 in case No. 635/6172/17. Available at: https://reyestr.court.gov.ua/Review/104728593.

^22^
See, in particular, the Decision of the Starobil district court of Luhansk region dated December 27, 2018 in case No. 431/4115/18. Available at: https://reyestr.court.gov.ua/Review/78392525.

^23^
Such cases are found in the Decision of the Holosiivsky District Court of the city of Kyiv dated August 23, 2016 in case No. 752/7929/16-ц (Available at: https://reyestr.court.gov.ua/Review/61275011), the Decision of the Primorsky District Court of Odesa from July 5, 2019 in case No. 522/20396/18 (Available at: https://reyestr.court.gov.ua/Review/82831857).

^24^
The Decision of the Sosniv District Court of Cherkasy dated October 21, 2022 in case No. 712/10119/20. Available at: https://reyestr.court.gov.ua/Review/107043791.

^25^
The Decision of the Zhovtenevyy District Court of Kryvyi Rih dated December 19, 2022 in case No. 212/3718/22. Available at: https://reyestr.court.gov.ua/Review/108199195.

^26^
The Decision of the Dnipro District Court of Cherkasy dated March 27, 2019 in case No. 711/17/19. Available at: https://reyestr.court.gov.ua/Review/81024789.

^27^
The Decision of the Slovyanskyi City and District Court of the Donetsk Region dated February 8, 2023 in case No. 243/2585/22. Available at: https://reyestr.court.gov.ua/Review/108839812.

^28^
The Decision of the Vinnytsia City Court of the Vinnytsia Region dated January 24, 2023 in case No. 127/12775/22. Available at: https://reyestr.court.gov.ua/Review/108672416.

^29^
The Decision of the Darnytsky District Court of Kyiv dated October 4, 2022 in case No. 753/15426/20. Available at: https://reyestr.court.gov.ua/Review/106915823.

^30^
The Decision of the Holosiivsky District Court of the city of Kyiv dated June 25, 2019 in case No. 752/11085/19. Available at: https://reyestr.court.gov.ua/Review/82615042.

^31^
The Resolution of the Supreme Court dated September 1, 2021 in case No. 754/10080/19. Available at: https://reyestr.court.gov.ua/Review/99425886.

^32^

The Resolution of the Supreme Court as part of the panel of judges of the Third Judicial Chamber of the Civil Court of Cassation dated April 14, 2022 in case No. 308/9708/19. Available at: https://reyestr.court.gov.ua/Review/104086064.

^33^

The Resolution of the Supreme Court as part of the panel of judges of the Third Judicial Chamber of the Civil Court of Cassation dated April 14, 2022 in case No. 308/9708/19. Available at: https://reyestr.court.gov.ua/Review/104086064.

^34^
See more details in the Resolution of the Supreme Court dated April 14, 2022 in case No. 308/9708/19 (Available at: https://reyestr.court.gov.ua/Review/104086064) and in the Resolution of the Supreme Court dated September 1, 2021 in case No. 754/10080/19 (Available at: https://reyestr.court.gov.ua/Review/99425886).

^35^
The Decision of the Uzhgorod City and District Court of the Zakarpatskyy Region dated February 2, 2021 in case No. 308/9708/19. Available at: https://reyestr.court.gov.ua/Review/94815876. For identical reasons, the Prydniprovskyi District Court of Cherkasy in the Decision of March 27, 2019 in case No. 711/17/19 also refused to satisfy the claim for damages.

^36^
It is about the Resolution of the Civil Court of Cassation of the Supreme Court dated May 13, 2020 in case No. 711/17/19 and the Resolution of the Civil Court of Cassation of the Supreme Court dated April 14, 2022 in case 308/9708/19.

^37^
The Decision of the Terniv district court of the city of Kryvyi Rih, Dnipropetrovsk region, dated June 8, 2022, in case No. 215/294/21. Available at: https://reyestr.court.gov.ua/Review/104683338.

^38^
The Decision of the Saksagan District Court of Kryvyi Rih, Dnipropetrovsk Region dated May 18, 2022 in case No. 214/5601/21. Available at: https://reyestr.court.gov.ua/Review/104420460.

^39^
The Resolution of the Dnipro Court of Appeal dated December 20, 2022 in case No. 217/5601/21. Available at: https://reyestr.court.gov.ua/Review/107988495.

^40^
The Resolution of the Supreme Court as part of the panel of judges of the Third Judicial Chamber of the Civil Court of Cassation dated April 14, 2022 in case No. 308/9708/19. Available at: https://reyestr.court.gov.ua/Review/104086064.

^41^
Now this Law has lost its validity on the basis of the Law of Ukraine ‘ *On Amendments to Certain Laws of Ukraine Regarding the Regulation of the Legal Regime in the Temporarily Occupied Territory*’ dated April 21, 2022 No. 2217-IX. Available at: https://zakon.rada.gov.ua/laws/show/2217-20#Text).

^42^
This is, among others, the Decision of the Starobil district court of the Luhansk region dated December 27, 2018 in case No. 431/4115/18, the Decision of the Holosiiv district court of the city of Kyiv dated August 23, 2016 in case No. 752/7929/16-ц, the Decision Ivano-Frankivsk City Court of Ivano-Frankivsk Region dated February 7, 2017 in case No. 344/15673/16-ц (Available at: https://reyestr.court.gov.ua/Review/64694889).

^43^
The Resolution of the Supreme Court as part of the panel of judges of the Third Judicial Chamber of the Civil Court of Cassation dated April 14, 2022 in case No. 308/9708/19. Available at: https://reyestr.court.gov.ua/Review/104086064. References are made to the specified legal acts, in particular, in the Resolution of the Supreme Court of May 18, 2022 in case No. 428/11673/19 (Available at: https://reyestr.court.gov.ua/Review/104635313), the Decision of the Dovgyntsivsky District Court of the city of Kryvyi Rih, Dnipropetrovsk Region in case No. 211/3600/20, the Decision of the Oktyabrskyi District Court of Poltava City dated February 7, 2023 in case No. 554/10776/22 (Available at: https://reyestr.court.gov.ua/Review/109225780).

^44^
The Decision of the European Court of Human Rights in the case of *Louisidou v. Republic of Turkey (28/07/1998).* Available at: https://hudoc.echr.coe.int/ukr#{“tabview”:[“document”],“itemid”:[“001-58201”]}.

^45^
The Decision of the European Court of Human Rights in the case of *Louisidou v. Republic of Turkey (28/07/1998).* Available at: https://hudoc.echr.coe.int/ukr#{“tabview”:[“document”],“itemid”:[“001-58201”]}.

^46^
The Decision of the European Court of Human Rights in the case of *Louisidou v. Republic of Turkey (28/07/1998).* Available at: https://hudoc.echr.coe.int/ukr#{“tabview”:[“document”],“itemid”:[“001-58201”]}. The use of this Decision of the European Court of Human Rights was observed in the Decision of the Dovgyntsiv District Court of the city of Kryvyi Rih, Dnipropetrovsk Region in case No. 211/3600/20, the Decision of the Starobil District Court of Luhansk Region dated December 27, 2018 in Case No. 431/4115/18, the Decision of Leninskyi Kirovograd District Court dated December 4, 2018 in case No. 405/6702/18, the Decision of the Holosiivskyi District Court of the city of Kyiv dated August 23, 2016 in case No. 752/7929/16-ц, the Decision of the Khmelnytskyi City District Court dated July 21, 2022 in case No. 686/26852/21. It should be noted that this list of cases is not exhaustive.

^47^
The Decision of the European Court of Human Rights in the case of *Khachukayev v. Russia (23/04/2009*). Available at: https://hudoc.echr.coe.int/ukr#{“fulltext”:[“Khachukayev”],“itemid”:[“001-92369”]}.

^48^
The Decision of the European Court of Human Rights in the case of *Sagayeva and Others v. Russia (08/12/2015).* Available at: https://hudoc.echr.coe.int/fre#{“itemid”:[“001-160943”]}.

^49^
The Decision of the European Court of Human Rights in the case of *Islamova v. Russia (30/04/2015).* Available at: https://hudoc.echr.coe.int/ukr#{“tabview”:[“document”],“itemid”:[“001-154025”]}.

^50^
The Decision of the European Court of Human Rights in the case of *Sultygov and Others v. Russia (09/10/2014).* Available at: https://hudoc.echr.coe.int/ukr#{“tabview”:[“document”],“itemid”:[“001-146775”]}.
