# Supplementary material for: War damages compensation: a case study on Ukraine
Source: F1000Res. 2023 Sep 29;12:1250. [Version 1] doi: 10.12688/f1000research.136162.1 (PMC10665606; doi:10.12688/f1000research.136162.1)
Supplement: Peer review history

# Reviewer report: Reviewer response for version 1

**DOI:** 10.5256/f1000research.149298.r221690

**Published:** 2023-11-22

**Authors:** Oksana Kaplina

**Affiliations:** 1 Criminal Procedure, Yaroslav Mudryi National Law University, Kharkiv, Ukraine

The article I evaluated is devoted to the important and complex topic of compensation for the damage caused by the war in Ukraine. First of all, I want to emphasize that the authors have well worked out the prerequisites of the study, emphasizing important details, such as the limitations of alternative methods of compensation in Ukraine, researching different concepts of compensation and their international definitions, which is extremely important for their application in the Ukrainian reality.

 It is worth noting the commendable citation of various literature that is relevant for this study - international and national legal acts, research by domestic and foreign authors, as well as a significant amount of judicial practice, which determines the applied nature of the study.

 Unfortunately, the article lacks a statistical representation of the results of the analysis of court decisions, in particular, the quantitative indicators of the affected persons in the percentage ratio. Such details would help the reader better understand the discussed issues. However, despite this, the article has a comprehensive approach, a logical division into parts, and a consistent presentation of the material.

 Another minor shortcoming is the lack of due attention to the extent of the damage caused and their monetary value, being limited only to the determination of the amounts to be recovered due to the moral damage caused to the victims. In my opinion, this is a fairly important criterion, especially for the victims themselves. However, taking into account the fact that the article is aimed at a research analysis of the legal aspects of compensation, and not its economic content, such omissions did not affect the overall quality of the article.

 The authors have highlighted enough key issues of compensation for damage that it is appropriate to discuss them in one article. However, in my opinion, researchers should consider in more detail the question of assigning responsibility to the state, which has a positive duty to protect its citizens and population. This issue may become promising for further research.

 In the article, the authors indicated several areas for further research, which remained unexplained due to the limitations of the scope of the article and the set goals. I agree that this study will be incomplete without further work in these directions.

 It is also worth noting that the research methodology corresponds to the selected type of publication on the F1000Research platform.

 Conclusions are clearly presented and correspond to all intermediate conclusions that were made during the study and do not contradict the main part. The tasks set at the beginning of the article have been achieved.

 This article may be useful to other researchers in search of answers to questions related to compensation for damage caused by armed aggression, and its practical part may be important in the development of appropriate compensation mechanisms by authorized persons. Also, this article has the potential to become a new turn in the scientific discussion on the existing problems of reparations and post-war reconstruction of Ukraine.

 Taking into account all the above-mentioned points and despite minor shortcomings, I believe that the article deserves to be approved, and there is no need for revision.

Is the case presented with sufficient detail to be useful for teaching or other practitioners?

Yes

Is the work clearly and accurately presented and does it cite the current literature?

Yes

If applicable, is the statistical analysis and its interpretation appropriate?

Not applicable

Are all the source data underlying the results available to ensure full reproducibility?

Yes

Are the conclusions drawn adequately supported by the results?

Yes

Is the background of the case’s history and progression described in sufficient detail?

Yes

Reviewer Expertise:

War damages, criminal procedure, prosecution, tribunals

I confirm that I have read this submission and believe that I have an appropriate level of expertise to confirm that it is of an acceptable scientific standard.

# Reviewer report: Reviewer response for version 1

**DOI:** 10.5256/f1000research.149298.r211327

**Published:** 2023-11-08

**Authors:** Mohamad Albakjaji

**Affiliations:** 1 Prince Sultan University, Riyadh, Riyadh Province, Saudi Arabia

The topic is very interesting. It will be the basis for additional research in the future. It will also be a starting point for the mechanisms that will determine how to compensate victims of Gross Violations of International Human Rights Law and Serious Violations of International Humanitarian Law.

 However, the reviewer would like to provide feedback in order to make the research of greater academic and practical value.

 In the introduction, the Author stated that: Resolution of the Council of Europe3 is foreseen (18) to create an international framework for compensating for any harm, loss, or injury caused. To realize this task, it was proposed the creation of an international register of damage, in cooperation with Ukraine and the EU member states aimed to keep records pertaining to evidence and claims regarding damage, loss, or injury suffered by individuals and entities, both natural and legal, in Ukraine.

 In this point, the author should mention the international mechanisms which is needed to implement such resolutions. As we may know, adopting rules and resolutions are not enough to resolve the dilemma. Such European resolutions will not be effective if it is not adopted at the international level, or at least clear mechanisms for compensation are globally established.

 The authors stated that: *As stated by the UN in the document Basic Principles and Guidelines on the Right to a Remedy and Reparation for Victims of Gross Violations of International Human Rights Law and Serious Violations of International Humanitarian Law 2005…etc*

 Here, there is no discussion of the mechanisms used at the international level through which affected countries can oblige aggressor countries to pay compensation for damages.

 Yes, it is true that international law and international humanitarian law stipulate, *‘Restitution should, whenever possible, restore the victim to the original situation before the gross violations of international human rights law or serious violations of international humanitarian law occurred. Restitution includes, as appropriate: restoration of liberty, enjoyment of human rights, identity, family life and citizenship, return to one’s place of residence, restoration of employment and return of property’ (authors’ italics)*

*Also, the authors stated that: In particular, it is worth recalling that, according to the report, states should, among other things, make efforts to develop procedures that would allow groups of victims to submit claims for reparations and receive reparations, if appropriate.*

**

 The authors mentioned that countries must not only prosecute criminals, but must provide fair compensation to the victims as well. However, the author did not mention what methods of compensation should be resorted to at the international level. For example, the authors mentioned that the concept of compensation is mentioned in the Ukrainian Civil Code, Commercial Code, Property Rights, and other local laws. But the author did not address the concept of compensation and its elements in accordance with international law. This is very important because the conflict between Russia and Ukraine is an issue that crosses the internal borders of countries, and therefore international mechanisms must be resorted to understand how to compensate for damages.

 Who can apply for compensation (reimbursement)?

*The author stated that, the Ukrainian legal field does not yet have a compensation mechanism for damages caused by armed aggression to business entities.*

 So as long as the local legal frameworks do not have yet a compensation mechanism, so it is so important to discuss the mechanisms victims may use internationally. The authors should discuss it.

 Although the writer mentioned most of the categories of plaintiffs who are entitled to compensation, the article needs a deep analysis regarding the rights of these categories and whether they will be able, in practical terms, to obtain fair compensation

 We also find that the article lacks in-depth analysis. The article is a descriptive enumeration of the categories of people who are entitled to compensation. It would have been better to write using both descriptive and analytical methods. For example, when the categories entitled to compensation were determined, the possibility of obtaining this right from a practical standpoint was not discussed, and what are the difficulties that those seeking compensation will face in obtaining compensation, whether these difficulties are related to legal challenges or the lack of competent institutions to assess damages or determine the amount of damage. Which affects every person or group

 Who is responsible for compensation?

 In general, this issue has been discussed from a theoretical perspective only. It has been determined how the Ukrainian government will approach the Russian government for compensation. The foundations of liability were also discussed, such as harmful action, damage, and causal relationship.

 But it would also have been better for the writer to discuss the issue of forcing Russia to pay compensation, what international legal frameworks can be used to demand Russia to pay compensation, and how effective such means are. All of these points must be discussed in order to have a complete picture of all the issues related to the extent of Russia’s responsibility for war damage and how to demand it to pay compensation, etc

 To make the research stronger, the researchers need to clarify what are the grounds Ukraine may relay on when claiming for compensation. They need to conduct acritical analysis of the main rules used for this purpose. I suggest here to study the international rules specially the main conventions that Russia breaches. Also, the international warfare rules, and the roles of the international criminal course, and the international court of justice in this process should be studied.

Is the case presented with sufficient detail to be useful for teaching or other practitioners?

Yes

Is the work clearly and accurately presented and does it cite the current literature?

Yes

If applicable, is the statistical analysis and its interpretation appropriate?

Not applicable

Are all the source data underlying the results available to ensure full reproducibility?

Yes

Are the conclusions drawn adequately supported by the results?

Yes

Is the background of the case’s history and progression described in sufficient detail?

Partly

Reviewer Expertise:

International Environmental Law

I confirm that I have read this submission and believe that I have an appropriate level of expertise to confirm that it is of an acceptable scientific standard.

## References

[1] : THE RESPONSIBILITY FOR ENVIRONMENTAL DAMAGES DURING ARMED CONFLICTS: THE CASE OF THE WAR BETWEEN RUSSIA AND UKRAINE. *Access to Justice in Eastern Europe* .2022;5(4-2) : 10.33327/AJEE-18-5.4-a000444 82-101 10.33327/AJEE-18-5.4-a000444
